# A qualitative analysis of young adults’ beliefs about bullying: exploring associations with social anxiety and post-traumatic stress

**DOI:** 10.1080/20008066.2025.2523638

**Published:** 2025-07-18

**Authors:** Belinda Graham, Anke Ehlers

**Affiliations:** a Department of Psychology, Institute of Psychiatry, Psychology and Neuroscience, King’s College London, London, UK; b Department of Experimental Psychology, University of Oxford, Oxford, UK

**Keywords:** Bullying, beliefs, cognitive behavioural therapy, social anxiety, PTSD, Bullying, creencias, terapia cognitivo conductual, ansiedad social, TEPT

## Abstract

**Background:** Bullying can be associated with emotional and social difficulties, but not all individuals experience enduring negative effects.

**Objective:** This study aimed to explore beliefs about bullying, self, and other people among young adults who were bullied that may be associated with ongoing anxiety and distress related to those experiences.

**Method:** Semi-structured interviews with 20 people, aged 18–29 years, who had experienced bullying were analysed using thematic analysis. The sample was split, by current symptoms of social anxiety and post-traumatic stress related to bullying, into a lower symptoms group (*n* = 12) and a higher symptoms group (*n* = 8).

**Results:** Participants reported multiple types of bullying, including online. Four superordinate themes were identified in negative beliefs related to bullying experiences: personal deficiency (i.e. victimization was due to own low value or undesirable traits), social threat (i.e. wariness of others due to their negative motives or traits), acceptance is fragile (i.e. being accepted by others is transient and requires effort), and minimizing (i.e. downplaying severity and impact of past experiences). These were evident in both groups but were more frequently endorsed in the higher symptoms group.

**Conclusion:** Negative appraisals related to bullying can persist into young adulthood and may influence social interactions and mental health. Interventions targeting these beliefs could mitigate negative outcomes and bolster resilience among individuals affected by bullying. Further research should explore these themes to inform effective therapeutic strategies for young adults who have been bullied.

## HIGHLIGHTS

- This paper explores negative beliefs about bullying, self, and other people among young adults who were bullied.
- Themes were identified in negative beliefs related to bullying experiences that were common across the sample and elevated among those with higher social anxiety or post-traumatic stress symptoms related to bullying.
- Negative appraisals related to bullying can persist into young adulthood, and interventions targeting these beliefs could mitigate negative outcomes.

## Abstract (es)

**Antecedentes:** Bullying se asocia a un mayor riesgo de dificultades sociales y emocionales entre los jóvenes que pueden persistir hasta la edad adulta, pero las consecuencias negativas a largo plazo no son universales.

**Objetivo:** Este estudio tuvo como objetivo explorar las creencias acerca del bullying, sobre sí mismo, y sobre otras personas en adultos jóvenes que sufrieron de bullying y que pudieran estar asociadas con ansiedad y malestar actual relacionadas con esas experiencias.

**Método:** Se analizaron entrevistas semiestructuradas a veinte personas, con edades 18 a 29, que habían experimentado bullying mediante análisis temático. La muestra se dividió, según los síntomas actuales de trastorno de ansiedad social y trastorno de estrés postraumático relacionado al bullying, en un grupo con síntomas más leves (*n *= 12) y en un grupo con síntomas más graves (*n* = 8).

**Resultados:** Los participantes reportaron múltiples tipos de bullying, incluyendo acoso en línea. Se identificaron cuatro temas principales en las creencias negativas relacionadas con las experiencias de bullying: deficiencia personal (ej., la victimización se debió a la propia baja autoestima o rasgos indeseables); amenaza social (ej., desconfianza hacia los demás debido a sus motivos o rasgos negativos); la aceptación es frágil (ej., ser aceptado por los demás es transitorio y requiere esfuerzo); minimización (ej., minimizar la severidad y el impacto de las experiencias pasadas). Estos temas fueron evidentes en ambos grupos, pero se manifestaron con mayor frecuencia en el grupo con síntomas más graves.

**Conclusión:** Las evaluaciones negativas relacionadas con el bullying pueden persistir hasta la adultez temprana y pueden influir en las interacciones sociales y la salud mental. Intervenciones que se centren en estas creencias podrían mitigar las consecuencias negativas y fortalecer la resiliencia de las personas afectadas por bullying. Se recomienda que futuras investigaciones exploren estos temas para fundamentar estrategias terapéuticas eficaces para los adultos jóvenes que han sufrido de bullying.

## 1. Introduction

Bullying is associated with an increased risk of social and emotional difficulties among young people that can persist into adulthood (Arseneault, 2018), but long-term negative consequences are not universal. Cognitive models suggest that maladaptive beliefs play a central role in the development and maintenance of anxiety or post-traumatic stress disorder (PTSD) following highly stressful events. Although such maladaptive beliefs differ widely between individuals, identifying common themes can help to explain specific problems and support clinical assessment and interventions. This paper presents qualitative analysis of interviews with young adults who were bullied, exploring beliefs about bullying, self, and other people across individuals with varying current distress related to their experiences.

Bullying is a form of interpersonal aggression that is so pervasive that it was historically dismissed as a normative part of growing up. However, since pioneering work in Scandinavia in the 1970s that recognized the potential harm and risks associated with bullying (Olweus, 1978), awareness has steadily increased. Bullying is difficult to define and conceptualizations have evolved over time (Volk et al., 2014), but it is frequently understood to include intentional and repetitive interpersonal aggression in the context of a power imbalance in favour of the perpetrator. Intentionality implies that the behaviour is deliberately hurtful. Frequency distinguishes bullying from one-off attacks. The presence of a power differential sets bullying apart from disagreements, teasing between friends, playful fighting, or general hostility, and is of particular importance given the broad potential implications of abuses of power for individuals and society (Andrews et al., 2023). International surveys indicate that up to 45% of children experience bullying at some point (Craig et al., 2009; Skrzypiec et al., 2018), although prevalence estimates vary significantly across countries (Cosma et al., 2020). Rates of cyberbullying, where bullying takes place online, have risen globally, with estimates ranging from 14% to 57.5% (Zhu et al., 2021), and online and offline bullying frequently co-occur (Li et al., 2024). With such high prevalence, it is a priority to understand more deeply the ways in which bullying may impact young people as they transition out of school into adulthood.

Adults who suffer with social anxiety disorder (SAD), typified by excessive anxiety about social situations in which they may feel judged by other people, are particularly likely to report having been bullied when they were younger (Boulton, 2013; McCabe et al., 2010). Several studies (e.g. Idsoe et al., 2021) have demonstrated associations between bullying and symptoms of PTSD, which include intrusive recollections of aspects of the bullying events, avoidance of internal or external reminders, negative cognitions and affect, and hypervigilance. Experiences that do not involve physical violence do not strictly fit the Diagnostic and Statistical Manual of Mental Disorders, Fifth Edition (DSM-5) or International Classification of Diseases, 11th revision (ICD-11) criteria for trauma that is a prerequisite for developing stress-related disorders, including PTSD (American Psychiatric Association, 2013; World Health Organization, 2019). However, extremely unpleasant social events during which an individual experiences intense anxiety and perceives ridicule or rejection by others have been defined as socially traumatic (Wild & Clark, 2011). These types of events occur particularly frequently and repetitively in the context of bullying or victimization by peers and, given the developmental context and repetitive nature of experiences, bullying is a particularly potent context for social trauma. Therefore, social anxiety and post-trauma symptoms are of particular interest among people who were bullied. There is a gap across research and clinical practice in conceptualizations of bullying and interpersonal or social trauma (e.g. Idsoe et al., 2021) but, given the known risks of bullying to mental health, there is clear urgency to bridge the gap to incorporate these experiences within clinical assessment and treatment.

Existing qualitative studies have produced insights into individual and interpersonal factors relevant to persistent difficulties related to bullying. Thornberg et al. (2013) conducted interviews with young adults who were bullied and suggested that internal victimization appeared to endure after the bullying stopped. They identified relevant linked cognitions and behaviours; namely, distrust of others, a sense of not fitting in, and self-doubt, with attempts at self-protection such as withdrawing socially, appearing emotionally unconcerned, and attempting to blend in or become socially invisible. These findings echoed an earlier study with men who were bullied as children (Carlisle & Rofes, 2007), who attributed significant long-term negative effects to their experiences, such as relationship difficulties, elevated fear and anxiety, feelings of isolation, shame, and thoughts of revenge. Similarly, emerging adults with poor mental health who perceived themselves as suffering long-term consequences of victimization (Lidberg et al., 2022) reported persistent feelings of insecurity and worthlessness, as well as avoidance of social situations. Victims of cyberbullying (Pabian & Vandebosch, 2021) reported similar long-term effects, including increased anxiety, poorer self-esteem, greater empathy, and relational challenges such as reluctance to share personal information and difficulty managing conflicts. Concerns about weight and body image have also been reported (deLara, 2019; Lidberg et al., 2022). This study adds to this literature by exploring beliefs related to bullying as they may relate to social anxiety and post-trauma symptoms, and identifying common themes with a view to supporting clinical assessment and interventions.

This article presents a primary qualitative analysis of interviews with 20 young people aged 18–29 years who reported a history of bullying. In addition to describing the nature of the bullying and how it is remembered, the primary aim of this analysis is to identify young people’s beliefs about themselves and others in relation to those experiences. We hypothesized that unhelpful beliefs about self and others related to past bullying experiences may contribute towards anxiety that persists even in the absence of current threat; that is, even after the bullying has stopped. Therefore, beliefs were explored among individuals with higher and lower current symptoms of social anxiety and/or PTSD related to bullying.

## 2. Method

An online questionnaire study about bullying and mental health was advertised via student newsletters and free online message boards, targeting young people aged 18–29 years. This period of adulthood (variously qualified as early, emerging, or young) was specified owing to its being proximal to childhood and adolescence, when bullying experiences commonly occur, discrete from late adolescence and later adulthood, and relatively uniform as a cohort compared with including participants from across the lifespan. After completing these questionnaires, participants were asked to tick a box if they were interested in meeting to discuss their experiences further as part of an interview study. Out of 115 people who completed the questionnaires, a total of 59 expressed an interest in the interview and the first 20 people who wished to take part were interviewed in person by the first author (BG) at the research offices in Oxford and London, UK. The interviewer was a clinical psychologist in the UK who specializes in trauma and anxiety and has received training in qualitative methods. Participants gave their written informed consent. Semi-structured interviews (see supplementary materials) lasted for 60–90 min, after which participants were debriefed and offered information about resources and support services relevant to their experiences. They were compensated with shopping vouchers. Audio files were transcribed, excluding identifiers, and then deleted. The study received institutional ethical approval [R51437/RE001]. Data were collected between 2017 and 2018, prior to the coronavirus disease 2019 (COVID-19) pandemic.

### 2.1. Inclusion and exclusion criteria

Participants were English speaking, aged 18–29 years, and reported, via an online questionnaire, that they had at least one experience of being picked on by peers that included a negative power differential (described in Section 2.2). Participants’ characteristics are shown in Table 1.

**Table 1.** Demographics, bullying victimization characteristics, symptoms of social anxiety disorder, and post-traumatic stress related to bullying.

	Lower SAD and/or PTSD symptoms related to bullying	Higher SAD and/or PTSD symptoms related to bullying	Statistic (*t*, *χ^2^*)
	(*n* = 12)	(*n* = 8)	
Demographics			
Age (years), mean (*SD*)	23.50 (2.88)	23.00 (3.63)	*t* = .344
Gender, *n* (%)			
Female	9 (75.0)	8 (100.0)	*χ^2^* = 2.353
Male	2 (16.7)	0 (0)	
Other	1 (8.3)	0 (0)	
Sexual orientation, *n* (%)			
Heterosexual	5 (45.5)	7 (87.5)	*χ^2^* = 3.753
Bisexual	4 (36.4)	1 (12.5)	
Homosexual	1 (9.1)	0 (0)	
Unsure/prefer not to say	2 (16.7)	0 (0)	
Ethnicity, white, *n* (%)	7 (58.3)	5 (62.5)	*χ^2^* = 2.112
Current employment, *n* (%)			
Student	8 (66.7)	3 (37.5)	*χ^2^* = 3.766
Employed	4 (33.3)	4 (50.0)	
Unemployed	0 (0.0)	1 (12.5)	
Victimization characteristics			
Total number of types of bullying, mean (*SD*)	5.58 (1.44)	6.25 (1.83)	*t* = −0.910
Types of bullying experienced, *n* (%)			
Teased	11 (91.7)	8 (100.0)	
Gossiped about	11 (91.7)	8 (100.0)	
Ignored	10 (83.3)	7 (87.5)	
Hit	9 (75.0)	5 (62.5)	
Sexual comments	8 (66.6)	7 (87.5)	
Threatened	6 (50.0)	4 (50.0)	
Belongings stolen	5 (41.7)	6 (75.0)	
Online	5 (41.7)	3 (37.5)	
Other	2 (16.7)	2 (25.0)	
Frequency of worst bullying, *n* (%)			
A few times a year or less	2 (16.7)	1 (12.5)	
About once a month	2 (16.7)	0 (0)	
2 or 3 times a month	1 (8.3)	2 (25.0)	
About once a week	1 (8.3)	0 (0)	
Several times a week	6 (50.0)	5 (62.5)	
Social anxiety symptoms^a^, mean (*SD*)	22.67 (3.89)	36.86 (7.84)	*t* = −5.32*
PTSD symptoms^b^, mean (*SD*)	15.25 (7.78)	27.88 (21.15)	*t* = −1.62

Note: ^a^ Measured with the 17-item Social Phobia Inventory (SPIN; range 0–68) (Connor et al., 2000). ^b^Measured with the 20-item PTSD Checklist for DSM-5 (PCL-5; range 0–80) (Weathers et al., 2013). ‘Higher symptoms’ were defined as PCL-5 ≥ 32 and/or SPIN ≥ 31.

SAD = social anxiety disorder; PTSD = post-traumatic stress disorder.

*p < .001.

### 2.2. Measures

Participants completed questionnaires online via Qualtrics (qualtrics.com) before the interview. They reported experiences that happened to them ‘by peers in a mean or hurtful way’, using the California Bullying Victimization Scale adapted for adult self-report (CBVS-R) (Green et al., 2018), which includes categories of being ignored, being gossiped about, being teased, having belongings stolen, receiving sexual comments, being hit, being threatened, or experiencing online abuse. They selected their ‘worst’ experience (the one that ‘currently bothers you the most’). The existence of a power differential at the time was established by rating the ‘main person or people involved’ in the worst experience as more popular, intelligent, physically strong, attractive, athletic, wealthy, or older. They also reported the frequency at which they experienced the worst type of bullying (a few times a year or less, about once a month, two or three times a month, about once a week, or several times a week). Participants reported current PTSD symptoms using the 20-item PTSD Checklist for DSM-5 (PCL-5; range 0–80) (Weathers et al., 2013) and social anxiety symptoms using the 17-item Social Phobia Inventory (SPIN; range 0–68) (Connor et al., 2000). Participants also provided their age, gender, sexual orientation, employment status, and ethnicity.

#### 2.2.1. Semi-structured interview

The interview content and structure were developed based on literature on bullying consequences, discussions with experts in cognitive therapy for anxiety disorders, and piloting. Participants were asked about the nature of bullying they experienced, and their perceptions of how it may have impacted them, including their beliefs about self and others. There were additional questions based on memory and coping strategies.

### 2.3. Data analysis

A thematic analysis was chosen to explore patterns within and between interviews, with detailed methods provided for transparency (Braun & Clarke, 2021). The first author (BG) read and reread the transcriptions to familiarize herself with the data before coding using NVivo 12 (QSR International Pty Ltd 2018). BG created detailed codes reflecting personal perceptions of what was said in each interview, then developed themes describing patterns across interviews, clustering them into superordinate and subordinate themes. Initial themes were reviewed and discussed with the second author (AE). Once the primary coding and review had been completed, an independent rater coded 10% of statements into the previously defined themes (Barbour, 2001), achieving high reliability (kappa = 0.85). Inconsistencies were further discussed and themes refined once again. Themes are not exhaustive and the authors note their own positionality as white female clinical psychologists with a background in cognitive–behavioural approaches that may influence data gathering and interpretations. The importance of each theme is not necessarily equivalent to its prevalence, but the number of participants who made statements that were coded within each theme was recorded to provide one marker of relevance.

Themes were explored across the sample and between groups with higher or lower symptoms of social anxiety and PTSD related to bullying. Higher symptoms were defined as meeting criteria for probable diagnosis of PTSD related to bullying (PCL-5 score 32 or above; range 0–80) (National Collaborating Centre for Mental Health, 2024) and/or reporting social anxiety symptoms indicative of moderate or severe impairment (SPIN score 31 or above; range 0–68) (Connor et al., 2000). Note that a SPIN score of 19 indicates the presence of social anxiety (National Collaborating Centre for Mental Health, 2024), but a higher threshold was chosen given the high levels of social anxiety symptoms in the sample overall. Lower symptoms were defined as below these thresholds on both measures.

## 3. Results

### 3.1. Participants

Participants reported varying severity of SAD and PTSD related to bullying (Table 1). They were grouped into those with higher (*n* = 8) or lower (*n* = 12) symptoms of social anxiety and/or PTSD. Most participants were white female students with multiple bullying experiences. No significant differences were found in background characteristics or bullying experiences between the groups.

Frequencies of themes are presented in Table 2.

**Table 2.** Frequencies of superordinate and subordinate themes.

Superordinate theme	Frequency, *n* (%)		Subordinate theme	Frequency, *n* (%)	
	Low symptoms	High symptoms		Low symptoms	High symptoms
Bullying impacted my life	12 (100)	8 (100)	Damaged my mental health	12 (100)	8 (100)
			Positive consequences	5 (42)	4 (50)
Personal deficiency	10 (83)	8 (100)	I should not be liked	8 (67)	7 (88)
			I am worth less than others	6 (50)	6 (75)
			I deserve to be targeted	3 (25)	5 (63)
			I can’t judge how others perceive me	3 (25)	5 (63)
Social threat	6 (50)	7 (88)	Friendships are unsafe	4 (33)	5 (63)
			People cannot be trusted	5 (42)	3 (38)
Acceptance is fragile	7 (58)	7 (88)	Rejection is inevitable	6 (50)	6 (75)
			I need to blend in	3 (25)	3 (38)
			I need to be perfect	4 (33)	4 (50)
Minimizing	5 (42)	7 (88)	I should be over it	3 (25)	4 (50)
			It wasn’t bad enough to warrant a reaction	2 (17)	3 (38)

Note: Percentages reflect the proportion of participants in each group (low symptoms, *n* = 12; high symptoms, *n* = 8) who made a statement coded under that theme. High symptoms were defined as post-traumatic stress disorder related to bullying [PTSD Checklist for DSM-5 (PCL-5) ≥ 32] and/or social anxiety [Social Phobia Inventory (SPIN) ≥ 31].

### 3.2. Characteristics and impact of bullying

Participants reported a range of bullying experiences, including online and offline incidents, being ignored, being gossiped about, being teased, having belongings stolen, receiving sexual comments, being hit, and being threatened. For most participants, their ‘worst’ experience occurred during secondary school (ages 11–18). Lower and higher symptom groups did not differ in the types or frequency of bullying encountered.

I was like, physically terrified to go to school. I hated going to school. I would walk into, like, rooms and people just didn’t even bother to stop talking about me. They would just make fun of me right to my face. And it was terrible. [108]

Also cyberbullying from girls at my school sort of called me names and would always sort of leave comments on my profile, they’d be quite rude and mean. [113]

Chewing gum in the hair … I got locked in the change room quite a lot … And a small kick to the shin when I would ask to get out the class. A kick to shin if you tell anything. [111]

#### 3.2.1. Perceived impact of bullying

All participants reported a negative effect on their mental health during the bullying, while perceptions of lasting impact varied considerably, including being one of many potential contributing factors to issues such as anxiety and depression. Some noted that memories and emotions related to bullying were more prominent during periods of poorer mental health and may have exacerbated the negative impact of challenging events later in life. Many also mentioned facing additional life stresses at the time of the bullying, which either overshadowed or compounded its impact.

See at the same time my parents had broken up when I was 11 and … they were very unhappy with each other and arguing, passive aggressiveness in general in the house and I think that contributed to me maybe thinking that that was in some way my fault as well or that I deserved that as well. [112]

Because the other thing that I found difficult then was, I moved in with my mum and my step-dad, right … So, it's quite hard to tell. [115]

Several participants expressed feeling conflicted between different perspectives on bullying – whether it was seen as a minor issue or a serious problem by themselves or others. Some participants noted that their mental health improved when they acknowledged how deeply bullying had affected them. They believed that avoiding or downplaying the issue in the past had not been helpful and may have worsened their earlier struggles.

With verbal abuse, I think it’s much easier to kind of … To get away with it and it’s … Like it’s more difficult to see on the victim … Like, you know, you don’t see things written … Things that people have said about you … But if you saw a black eye you’d know someone’s been bullying that guy. [103]

Yes, and it took me until very recently to realize, wow, this period did matter. It feels like it’s so far back that it shouldn’t matter at all. But then kind of really giving myself permission to say that was a trauma in its own way and it had nothing to do with me. That gave me a sense of relief, I think. [116]

I started to realize, you know, actually maybe I feel low about myself because you know I’ve taken on the way they put it on me … I definitely wanted to deny that these people could have any impact on me whatsoever. I wasn’t going to allow it to affect me. But it had. [103]

Participants in both low and high symptom groups reflected on some aspects of personal growth related to bullying, including compassion for others, recognizing personal strength, and openness to expressing their individuality.

I feel like my compassion for other people is a lot more expansive because of it. [108]

Looking back now, the fact that like I was able to manage and get through all of the things shows that I’m quite a strong person. [103]

It made me feel like I was different but it also made me feel like I kind of had a licence be myself in a way. So, I felt like I, like I say kind of like accepted that I wasn’t kind of perfect or well liked. So, then it was sort of like well I’ll just wear what I want and say what I want. I can be weird because they already think I am. [113]

### 3.3. Beliefs about self and others

#### 3.3.1. Personal deficiency

Several participants made statements that suggest they believe they deserve to be at the bottom of a social hierarchy. They reported acceptance of being targeted and disliked because of personal deficiencies. They described their treatment as a reflection of their own low value or undesirable traits, rather than negative traits in others or external factors.

##### 3.3.1.1. I should not be liked

This subtheme refers to participants questioning others’ affection and whether they can genuinely feel positively about them despite perceived deficiencies, even within positive and close relationships.

Even with friendships, I still sometimes have the … thoughts where I’m like, oh, if he or she didn’t answer me back, it’s because I’m annoying them, I’m a not a good friend to them and everything. Five years later and I’m still working at that. So working and giving myself … I’m worth it. You know, building that confidence back and that’s hard. [111]

I think the thing that, like, still stays with me is feeling like I am intentionally being left out. Or, like, that the people are, like, engaging with me out of, like, politeness, rather than genuine interest in my life. [108]

##### 3.3.1.2. I am worth less than others

This subtheme refers to bullying driving or reinforcing beliefs about being inferior to other people.

I never felt worthless before the bullying. And, still today, the fear of not being enough for someone. For them to leave me because I’m not enough to have them to stay by my side. [111]

And I’d say it reinforced some beliefs I already had about myself. Thinking that I was … My self-worth being low and confidence. I think that those were things that already existed in my head. So I think it reinforced them a bit. [106]

I just assumed it must be something wrong with me. It was proof, or I took it as proof that these negative things I believed about myself were true. [105]

Feelings of worth were tied to perceived social status relative to peers, with two participants noting their acute awareness of social hierarchies and deliberately positioning themselves among those deemed ‘low value’ to enhance comfort and increase their potential to advance within that subset.

I guess I always feel quite uneasy in peer situations. And I feel … I find myself drawn to the outsider people, as opposed to the cool people. If I can see it. If I can see that that distinction exists in a group. [109]

If there’s someone who's like less attractive or there's someone who's clearly not as confident, I will gravitate to them. Because then I feel like I can help them I guess to join in, and I always want to be like an enabler for people to feel comfortable and feel confident in themselves. Because then I feel like I'm the one who's in authority and I'm the one who's the highest level in that situation. I feel like I can kind of take control of that a bit better. [118]

##### 3.3.1.3. I deserve to be targeted

This subtheme refers to feeling permanently marked as a potential target, based on a belief that future targeting is likely because of intrinsic personal factors, regardless of ongoing victimization.

Or I think if it happened again, maybe I wouldn’t think it is so random, if it is happening again. But I sort of, like, crack it up to being, like, just kind of drawing the, like, social short straw. Like, I don’t know, for whatever reason, you are the perfect storm of someone who is easy to pick on. [108]

Just that I have a very strong sense of not being … I think maybe a lot of children or people have this sense that there is something fundamentally not right about them and that’s the feeling which is one of the strongest that I’ve had with my experiences. Almost like there’s something naturally wrong with you [105]

One participant described anticipating and accepting maltreatment.

I do find that sometimes when people, like, don't treat me very well, or like, say things that aren't necessarily appropriate and stuff, I don't tend to realize. I kind of expect it, and don't … And I don't realize until afterwards. So I don't know. Yes, I think I kind of sometimes expect to be treated badly, and then don't, don't really … And don't really realize that I'm being treated badly when I am, because I just kind of expect it. Which isn't very good. [117]

##### 3.3.1.4. I can’t judge how others perceive me

This subtheme refers to a belief of being less able than other people to judge how others perceive them, leading to uncertainty in social situations. Participants also described focusing on potential cues and putting effort into deciphering others’ behaviours and reactions.

I blindly navigate. I feel like I have a really hard time perceiving how people perceive me. [108]

I find it really hard to know if I am being too clingy or too aloof, like, I find it really, really hard to find the balance because I didn’t know I can trust my reading of the situation. So I don’t actually know if I am too quiet or not, because sometimes I just, like, don’t speak or don’t go and talk to people. But I don’t know. [119]

#### 3.3.2. Social threat

This theme was developed from statements suggesting wariness of others due to their being unreliable and untrustworthy.

##### 3.3.2.1. Friendships are unsafe

This subtheme refers to uncertainty about others’ motives within friendships, including questioning their intentions and limiting meaningful social contacts to avoid potential betrayal or abandonment.

I feel like having a very small network is almost more beneficial. It’s sort of a numerical thing. Where I am like, well, if I lose all of these people at one time … I’m like, I would rather process, emotionally, only losing five close friends, or having five close friends hurt me, than 25. [108]

Not only, but I don’t like really telling people that I get to know a lot about me or my life because I feel like it’s not safe maybe to do that. Or otherwise they’re not going to be interested in it at all. [112]

The sense of lack of safety associated with friendship was more deeply described by a participant who explained that although bullying seems less prevalent among adult relationships the possibility remains for hurtful behaviour that is perceived in the same way as bullying.

It’s more confusing as an adult, than anything. Like, at least children, like, they are simple creatures. Like, they treat people like garbage. And that’s very, like, obvious when they invite you somewhere as a joke and they laugh. At least they’re laughing in your face. And I think, as an adult, I’d much rather have someone, like, laugh in my face, than, like, me not know if they’re laughing behind my back. So, I think it’s more just the, like, subtlety of adulthood that’s got me quite on edge, and kind of paranoid. [108]

##### 3.3.2.2. People cannot be trusted

This subtheme refers to drawing connections between experiences of being bullied in the past and current unwillingness to rely on others, invest in them, or trust them to have good intentions.

And I think that’s the only thing that I have really carried up until now … I was just always really anxious about if people were, like, if people were being truthful with me or, like, if it was all a joke. I don’t really know how you would describe that … I think it’s just, like, the ambiguity of it all is what makes me feel like this heightened sense of like unawareness. Or, like, suspicion I guess? [108]

#### 3.3.3. Acceptance is fragile

This theme was developed from statements suggesting a belief that acceptance by others is transient and requires effort.

##### 3.3.3.1. Rejection is inevitable

This subtheme refers to a belief that rejection by friends is inevitable, uncontrollable, and a constant risk, even in the context of positive interpersonal experiences. Rejection may be perceived even when it is not occurring, as described by the following participant.

I think even if, for the rest of my life, I, you know, only ever really had … very nice interpersonal experiences, I would always still think, how long is this going to last? And I can’t see how I could ever be completely convinced. Because sometimes I experience things that aren’t rejections, or things that would cause this feeling. They can be very … They can be quite neutral really, looking at them from the outside. But I might still … [109]

One participant who was bullied for several years as a teenager reported having recovered from this experience in her later adolescence as she developed different friendships and became more confident in personal qualities and likability. However, a recent move to a new environment led her to have familiar feelings of being rejected associated with the prior bullying she had been through.

Because I had kind of forgotten about all of the things that happened, until I’m starting to feel those feelings again. Like, I guess I really haven’t dealt with this as, like, I probably should have. Or maybe this wouldn’t be happening. So, maybe I should have dealt with it. So, yes, it’s a weird thing to feel that all over again. I mean it’s not that I feel like totally alone. Because I have friends I can talk to. It’s just sort of isolating. And I feel as isolated as I did in middle school. [108]

Connected to a fear of being rejected, a subset of participants spoke of needing frequent reassurance that their friendships and romantic relationships are valued.

I just always need that sort of reassurance that people haven't gone off me or know that they haven't taken this in the wrong way … I do feel like I need some sort of contact or clarification just to double-check that they still like me. [118]

I think that's the thing is like I need people to tell me that they want me around and people don't do that naturally and I know that but I don't ever want to be a pest and I think that's like my worst trait as an adult is that I don't want to pester people. [107]

##### 3.3.3.2. I need to blend in

This subtheme represents effort towards being accepted socially, and reduce the perceived risk of rejection, by remaining neutral.

I guess when you see what it can be like when people turn on you and do stuff like that, it does make you, as well, want to go under the radar and just not have that happen. Well, just not do anything that makes you really noticeable, good or bad. Just like cruise in that middle area. Because then if you’re out there again, it might bring other stuff back up. Or something else might happen. It’s just easier to just coast a bit. [106]

I might have something to say, but not want to draw attention to myself. I will not express an opinion that could be seen as controversial. Unless I was very, very comfortable with the audience. And so, I think I described earlier. I have this kind of neutral mask. That I can’t really stop doing. [109]

##### 3.3.3.3. I need to be perfect

This subtheme represents effort towards being acceptable to others and to themselves by striving for perfection. With others, this included developing deep and perfect connections with friends, being unconditionally nice to escape criticism, or seeming impressive. In relation to feeling acceptable within themselves, this included contributing vastly to charity work or excelling academically.

It’s like a perfectionism that I have to be the perfect friend, I have to be perfectly understanding, I want them to think that I’m the only person … I’m not the only person, but I want them to think that I’m someone who really gets them. [105]

I’d, kind of, like, have a value because I’m nice. And that like I might not be … because no one’s perfect and, you know, maybe I’m not really, really good at one thing or really, really good another but I’m really nice, I’m nice, I can be nice. And find value I suppose. [113]

I want people to think the bar is pretty high. I want people to think I'm the funniest. I want people to think I'm really confident. Yes, I kind of feel like if I'm not immediately likeable and confident and the best version of me then people will kind of think oh she was boring, won't bother seeing her again. [118]

[Interviewer] Okay. And, if you weren’t 100% academically, how would it feel?

It would be horrible. I can feel my stomach sinking thinking about it. Because I don’t know how to be with myself, unless I have those rules that I make up for myself in place. [105]

I think it became an obsession really because it was the only way that I felt, maybe good. I could feel, I was allowed to feel good about myself. It was like approved, you know, if you get a grade, it’s black and white and you can say, oh that’s good. [112]

The feeling of worthlessness, yes. Because since that time, I’m still making myself do things to feel like I’m worth something. I even did a humanitarian trip into an orphanage to help the orphanage but also selfish enough to make me feel helpful. To have a cause. And I … And I want to work with charities. To help those charities, of course, but also I’m doing something, I’m actually helping people, I’m worth something. [111]

#### 3.3.4. Minimizing

Participants in both groups expressed feeling ashamed of being bullied and wished to downplay these experiences, fearing that revealing their past could harm their current social well-being.

I definitely feel ashamed to come here and talk about it, because it’s admitting … Like I said, admitting a part of your life you’re not proud of because it was proved that you weren’t good enough … I think I didn’t want to admit to anyone now that I ever was in a position where I did feel like that. [105]

I am slightly worried that I’ll be associated with these things in a negative way as if it was like my fault. [113]

##### 3.3.4.1. I should be over it

This refers to a belief that as an adult they should have left behind their negative experiences growing up.

I blame myself because I can't seem to be able to get over it. Like, I realize that it wasn’t my fault that I was bullied, but I think that it's, sometimes I think that it's my fault, sadly myself for not being able to move on because I keep thinking about it and it's, I still let it affect me. [104]

That’s what’s frustrating about it. Is like how have you not found a way to get over this feeling? How are you still, like, thinking that everyone doesn’t like you and doesn’t want to be around you, when you have plenty of people back home that love you? And who want to be around you. [108]

I suppose the ideas and things that I picked up then, that that still shapes, to a very large extent, I think, how I behave now and how I interpret things, and I suppose still finding things stressful maybe when you shouldn’t be and finding that annoying. [114]

##### 3.3.4.2. It wasn’t bad enough to warrant a reaction

This subtheme refers to a belief that bullying experiences were not serious enough to justify their reaction.

Sometimes I feel like a senior drama queen or do you know, I tell myself it wasn’t that bad. I mean, other people, you know, get raped beaten and I don’t know what, that is horrible. But then at the same time I've got a feeling that I want just especially the people involved to acknowledge that it happened. [112]

And compared with all of that, like, somebody making jokes about you being a lesbian, it's just one more thing … It felt worse. It was sort of, like, not even, you weren’t really kind of legitimate to be upset about it. [115]

## 4. Discussion

This study explored young people’s experiences of bullying and related beliefs about themselves and other people. Consistent with other studies on emerging adults who were bullied (deLara, 2019; Lidberg et al., 2022; Pabian & Vandebosch, 2021), all participants reported negative impacts on their social and emotional well-being. Themes were identified across the sample, but those with higher symptoms of social anxiety or PTSD related to their experiences more commonly reported a sense of being inherently problematic in themselves, unsafe in friendships, and at risk of rejection. They also more frequently tended to minimize the impact of bullying and hide their experiences.

A sense of personal deficiency and feeling stuck at the bottom of a social hierarchy were common, especially in the higher symptoms group, suggesting that these appraisals impact mental health among young people who were bullied. Both groups experienced mistrust and insecurity in friendships, but it appears that individuals in the higher symptoms group endorsed beliefs reflecting a perception of risk to themselves from others more frequently. It may be that this was related to lower acceptance of ambiguity in relationships, such as certainty regarding other people’s motives and intentions to either maintain or end a relationship. Some participants reported mistrust towards close friends or partners despite lacking evidence of any current malintent. Notably, beliefs of personal deficiency and a high social threat often co-occurred, with participants feeling deserving of low status (e.g. expecting to be treated poorly) and wary of more socially powerful people who might be more likely to inflict harm. Experiences of bullying may have led to overgeneralized negative beliefs about the self and contributed towards a sense of identity as inferior or deserving to be low in the social hierarchy.

Participants in both groups described striving for social acceptance, including trying hard to blend in and putting a lot of effort into trying to be perfect. Although this was reported in both groups, participants in the higher symptoms group seemed to subscribe more readily to the idea that, ultimately, rejection was inevitable, regardless of their best efforts to avoid this outcome. Efforts could include trying hard not to upset other people, maintaining a neutral stance, and being unquestionably nice or impressive. While these are not necessarily negative behaviours, they may be limiting if they disregard personal needs, and may reflect individuals continuing to act in ways that were adaptive when bullied but are no longer appropriate.

Minimizing past bullying experiences was particularly common among the higher symptoms group. Several participants in both groups expressed some shame about past experiences of being bullied, fearing that it would harm their current social well-being if others found out. They described a belief that past bullying indicated something negative about them that would remain problematic today, should it become known that they were bullied. There is therefore a wish to keep the information hidden. For example, if being a target of bullying holds a personal meaning of being weak or unattractive, then a person may fear that a similar interpretation could be made by current friends or acquaintances should they become aware of it. Participants described denying or downplaying the seriousness of bullying, despite acknowledging its negative impact on their lives.

Clinically, this exploratory work suggests specific cognitive themes that may be particularly relevant to the development and maintenance of anxiety among people who were bullied. Asking about bullying experiences and assessing cognitions linked to these themes could improve the specificity of clinical assessments and point towards targets for individualized interventions as part of cognitive therapy. Cognitive restructuring with methods recommended in cognitive therapy, such as surveys (Murray et al., 2022) or behavioural experiments (Murray & El-Leithy, 2021), could be used to address negative beliefs linked with these themes and test out predictions, including about social interactions or personal reactions. For example, a survey could be used to address a belief connected with the theme of minimizing, such as ‘people will think I am weak if they find out I was bullied’, that may include questions such as: ‘If you heard that a person was bullied in the past, what would you think? How much would you think it meant the person was weak, on a scale of 0–100%?’ As part of cognitive therapy, behavioural experiments could be set up to test predictions; for example, related to beliefs about negative consequences of appearing imperfect or standing out.

Future research could assess the extent to which beliefs aligned with these themes may predict later social anxiety or post-trauma symptoms. This is relevant for identifying people who may be at risk of developing problems and generating targets for early interventions. Future research could also explore these themes in relation to school-based bullying prevention efforts that could emphasize that victims are not to blame for their experiences and address negative appraisals at a systemic level. The authors noted that questions about romantic and intimate relationships were not directly posed in this research, but issues related to emotional and physical intimacy were raised by participants during the interviews. This included individuals questioning whether past difficulties in peer relationships may be relevant to their beliefs and behaviours within intimate relationships, such as excessive or inhibited closeness, and even revictimization. These topics could be addressed in future research.

### 4.1. Limitations

Interviews were developed from a cognitive behavioural perspective, but delivery was flexible and individualized to increase scope. The study used convenience sampling, and while the restricted age range is a strength (Lidberg et al., 2022), several factors may present a challenge to the generalizability of the findings. Groups of individuals with higher and lower symptoms were identified, but high levels of social anxiety were reported across the sample. The majority of participants identified as female and there was limited gender diversity across the groups, so additional investigations among gender-diverse individuals with high symptoms would be helpful. Of note, reported sexual orientation was mixed across the overall sample, but the diversity of sexual orientation was low in the higher symptoms group. Beliefs associated with bullying towards other people were not explored within these interviews, and including this focus in future work could introduce additional important perspectives.

## 5. Conclusion

This qualitative study explores appraisals among people who were bullied, highlighting similarities and differences between those with higher or lower current symptoms of social anxiety and/or PTSD related to past bullying experiences. Our results suggest significant short- and long-term impacts of bullying across the sample. However, negative beliefs about self and others, represented in the themes of personal deficiency, social threat, fragile acceptance, and minimizing, were more commonly reported in the higher symptoms group. Further exploration and testing of these themes could help to improve targeted interventions.

## Supplementary Material

Supplemental Material

## Data Availability

This research was based on detailed interviews with participants, who agreed for selected quotations, without identifying information, to be shared in the manuscript. To maintain confidentiality, additional supporting data are not available.
