# Supplementary material for: A qualitative analysis of young adults’ beliefs about bullying: exploring associations with social anxiety and post-traumatic stress
Source: Eur J Psychotraumatol. 2025 Jul 18;16(1):2523638. doi: 10.1080/20008066.2025.2523638 (PMC12278457; doi:10.1080/20008066.2025.2523638)
Supplement: Supplemental Material [file ZEPT_A_2523638_SM0216.docx]

What happened

Based on the questionnaire you completed, I can see that you had negative experiences with peers. I thought it would be helpful to have a shared term for this. What word/s or term/s do you use to describe what has happened to you?

- *Agree shared term for *bullying/teasing/being picked on/other*

It would be helpful to get a sense of when your experiences took place and what happened.

*Annotate timeline together.*

For each event/period:

What happened?

- *Direct/overt: name-calling, threats, verbal aggression, physical aggression*
- *Indirect/relational: used their status to harm me socially, exclusion, gossip*
- *Online/in person*

What age did it start? How long did it last? Is it ongoing?

- How severe was it? (0 – 10, least to most severe).
- Who was it? *Peers, sibling, authority figure, same/other group, someone else.*
- How frequently did it happen?
- Was it intentional?
- Were they more popular, stronger, smarter than you?

Looking over the timeline, what was the worst

- type of bullying*
- single event/period of time

Can you describe it to me?

- Feel free to describe even the worst moments if you are comfortable to do so. Include what you were feeling and thinking at the time.

Appraisals and impact “then”

Cast your mind back to the worst event or period that you described just now. At that time…

…what emotions did you feel?

- *Fear, shame, anger, sadness, humiliation, embarrassment…*
- *What was worst and what made it so bad?*

…what did you feel in your body, physical problems?

- Headache, pain, sleep problems, tiredness, lethargy, tingling, restlessness, other.

Beliefs about self

…what did it mean to you about yourself as a person that you were being bullied*?

- *What did you feel/think about yourself?*
- *What would other people say about you as a person?*

Beliefs about social self

…what did it mean to you about your relationships that you were bullied*?

- *What did you feel/think about your relationships with others?*
- *What would other people say about your relationships?*

Beliefs about others

…what did it mean to you about other people in general that you were being bullied*?

- *What did you feel/think about other people?*
- *What would other people say about your position in society?*
- *What did you feel about your position in society?*
- *How society functions and relationships work?*

Beliefs about blame

…whose fault did you think it was that you were being bullied*?

- *How much was it your fault (0-100)*
- *Why? What about you, your situation, your characteristics?*

…what did you do to cope?

- *Avoidance (e.g., specific types of social situations), alcohol or drugs, online gaming*
- *Social behaviour or ways of interacting, openness, other*

…did you talk about it with others (peers, authorities, family, other important person)?

- If so, how did they react? What did they say or do?
- If not, why not? What would have happened if you had told someone else?

Appraisals and impact “now”

Do you feel that you are continuing to experience difficulties as a result of being bullied*?

If yes, what difficulties are connected to what happened to you?

- *Anxiety, depression, work, relationships, appearance, other.*

If not, what do you think protected you or help you move on?

Beliefs about self

…NOW what does it mean about you as a person that you were bullied*?

- *…and have recovered from that experience/continue to suffer*

Beliefs about social self

…NOW what did it mean to you about your relationships that you were bullied*?

- *What does it make you feel/think about your relationships with others?*
- *What would other people say about your relationships?*

Beliefs about others

…NOW what does your experience of being *bullied** tell you about other people in general

- *What does it make you feel/think about other people?*
- *What would other people say about your position in society?*
- *What do you think about your position in society?*

Beliefs about blame

…NOW whose fault did you think it was that you were bullied*?

- *How much was it your fault (0-100)*

Looking back, what do you think is the reason you were *bullied**?

- *Was it something about you? (e.g., race, appearance, sexuality, something ‘different’)*
- *Was it something about the bully* or context? (e.g., mental health, family, others, etc)*

Does it matter that you were bullied?

Memories*

Do memories of bullying* ever pop into your mind?

- Can you think of a recent time?
- Can you describe what was happening? Was it words, image, video? Whose perspective?
- What triggered it? *An internal feeling (anxiety?), a social situation (someone who reminded you of…, familiar or unfamiliar person, dating or intimate, other), other*

Do you ever find yourself suddenly feeling like when you were bullied*? How do you feel?

- Can you think of a recent time?
- What triggered it?
- As above.

If memory or felt sense happened,

**…**what emotions did you feel?

- Fear, shame, anger, sadness, humiliation, embarrassment…
- What was the worst feeling you had and what made it so bad?

**…**what did you feel in your body?

…what did you do?

Coping strategies “then”

In the past, did you do anything to try to cope with effects of bullying* or prevent it from happening again? What kinds of things did you do?

- *Staying quiet, avoidance (conflict or expressing opinions, going where bullies* might be, online interactions, risk-taking in romance, work, social), saying yes to everything, rehearsing for social interactions in advance, being funny or smiling a lot, mental strategies like imagining things, other.*

Did you do things to help you manage situations (like social situations) back then?

Were there things that you did to make yourself feel better when it was especially hard?

- *Avoidance (e.g., specific types of social situations), alcohol or drugs, online gaming, TV, hobbies*
- *Social behaviour or ways of interacting, openness, other*

Were you ever involved in bullying* other people?

- *Yourself, assisting/reinforcing another bully*, witness, or defending others?*
- In person/online?

How did this make you feel?

Coping strategies “now”

Is there anything you do now to try to prevent bullying* from happening?

Or prevent feeling like you did when it happened?

Are there things you do to try to manage situations (like social situations) now?

Are there things you do to try to make yourself feel better now?

Rumination

Do you find yourself going round and round in your mind over the *bullying** you experienced? What is mostly in your mind when that happens?

- *How you could have prevented it from happening or made it less bad.*
- *How unfair it is?*
- *How the experiences impacted your life?*
- *What the bully* is doing now?*
- *Is there any justice?*
- *What if…*
- *If only…*

Is there anything you stew on or repeat over and again regarding the bullying*?

When you have the thoughts going round and round in your mind what emotions do you feel?

- *Fear, shame, anger, sadness, relief, shame, embarrassment*

When you are thinking about what happened, what do you feel in your body?

When something goes wrong or you make a mistake, what kinds of things do you say to yourself?

- Does the voice remind you of anyone? *Tone, words, experience.*
